# Cholesterol imbalance and neurotransmission defects in neurodegeneration

**DOI:** 10.1038/s12276-024-01273-4

**Published:** 2024-08-01

**Authors:** Kyung Chul Shin, Houda Yasmine Ali Moussa, Yongsoo Park

**Affiliations:** 1 Neurological Disorders Research Center, Qatar Biomedical Research Institute (QBRI), Hamad Bin Khalifa University (HBKU), Qatar Foundation, Doha, Qatar; 2 College of Health & Life Sciences (CHLS), Hamad Bin Khalifa University (HBKU), Qatar Foundation, Doha, Qatar

**Keywords:** Synaptic vesicle exocytosis, Neurodegeneration

## Abstract

The brain contains the highest concentration of cholesterol in the human body, which emphasizes the importance of cholesterol in brain physiology. Cholesterol is involved in neurogenesis and synaptogenesis, and age-related reductions in cholesterol levels can lead to synaptic loss and impaired synaptic plasticity, which potentially contribute to neurodegeneration. The maintenance of cholesterol homeostasis in the neuronal plasma membrane is essential for normal brain function, and imbalances in cholesterol distribution are associated with various neurodegenerative disorders, including Alzheimer’s disease, Parkinson’s disease, and Huntington’s disease. This review aims to explore the molecular and pathological mechanisms by which cholesterol imbalance can lead to neurotransmission defects and neurodegeneration, focusing on four key mechanisms: (1) synaptic dysfunction, (2) alterations in membrane structure and protein clustering, (3) oligomers of amyloid beta (Aβ) protein, and (4) α-synuclein aggregation.

## Exploring Cholesterol Imbalance: Link to Neurodegenerative Diseases

Cholesterol, a substance crucial for the brain, can lead to diseases like Alzheimer’s and Parkinson’s when imbalanced. This review investigates how this imbalance causes brain cell degeneration, focusing on issues like communication breakdown and harmful protein build-up. The study combines findings from different experiments to understand cholesterol’s role in the brain. The review emphasizes the need for cholesterol balance for brain health and identifies potential treatment targets for neurodegenerative diseases. The main findings suggest that cholesterol imbalance disrupts brain cell communication and leads to harmful protein build-up, causing brain cell degeneration. The researchers conclude that focusing on cholesterol metabolism and distribution could lead to new treatments for these conditions. Future research may lead to treatments that correct cholesterol imbalances, possibly slowing or preventing neurodegenerative diseases.

This summary was initially drafted using artificial intelligence, then revised and fact-checked by the author.

## Introduction

Cholesterol is a lipid that is critical for the structure and function of cell membranes in the brain, where it participates in neuronal signaling and synaptic transmission. The brain contains the highest concentration of cholesterol in the human body, accounting for 20–25% of the total cholesterol^1,2^. The blood–brain barrier (BBB) is impermeable to peripheral cholesterol^3^; therefore, most cholesterol in the brain is generated by de novo synthesis, mainly in the glia and to a lesser extent in neurons^3^. The high cholesterol concentration suggests an important role for cholesterol in brain physiology.

Cholesterol is involved in neurogenesis and synaptogenesis^4,5^. Age-related reductions in cholesterol levels in the plasma membrane lead to synaptic loss^6,7^ and impaired synaptic plasticity^8^, suggesting that cholesterol imbalance in the neuronal plasma membrane affects neuronal activity and contributes to neuronal degeneration^9,10^. The maintenance of cholesterol homeostasis is essential for normal brain functions^11–13^. An imbalance in cholesterol distribution can cause the pathological changes observed in various neurodegenerative diseases, such as Alzheimer’s disease (AD), Parkinson’s disease (PD), and Huntington’s disease (HD), suggesting that neurodegenerative diseases are associated with dysregulated cholesterol distribution^11,12,14^.

The aim of this review is to examine the molecular and pathological mechanisms by which cholesterol imbalance causes neurotransmission defects. This review focuses on four molecular mechanisms to explain how cholesterol imbalance in neurons results in neurodegeneration: (1) synaptic dysfunction, (2) membrane structure and protein clustering, (3) amyloid beta (Aβ) aggregation, and (4) α-synuclein (α-syn) aggregation.

## Main Text

### The molecular mechanisms of neurodegeneration induced by cholesterol imbalance

Cholesterol has a complex and multifaceted role in neurodegeneration. Given that cholesterol is an essential component of cell membranes and is involved in various physiological processes in the brain, an imbalance and dysregulation of cholesterol homeostasis can contribute to the pathogenesis of neurodegenerative diseases^11,12,14^. Table 1 summarizes the links between cholesterol and different neurodegenerative diseases. Several mechanisms have been proposed to explain how cholesterol imbalance may contribute to neurodegeneration:

1. Synaptic dysfunction: cholesterol is critical for the formation and function of synapses, the connections between neurons that facilitate communication in the brain. Altered cholesterol levels can affect synaptic transmission and plasticity, impairing neuronal signaling and contributing to the cognitive deficits observed in neurodegenerative diseases. The plasma membrane is enriched with cholesterol, ~80% of which is cellular cholesterol^15^. Therefore, reduced cholesterol levels in the plasma membrane, i.e., cholesterol imbalance, impairs synaptic transmission and plasticity and thus induces neurodegeneration^9,10,16^.
  Depletion and imbalance of cholesterol in the plasma membrane cause deficits in neurotransmission; e.g., cholesterol depletion reduces Ca^2+^-dependent exocytosis of large dense-core vesicles (LDCVs)^17^, cortical secretory vesicles^18^, and synaptic vesicles in hippocampal neurons^9,19^, cortical synaptosomes^20^, ribbon synapses^21^, and motor nerve terminals^22^. However, unveiling the molecular pathology of cholesterol imbalance in neurodegeneration is challenging because cholesterol is involved in various cellular signaling processes and neuronal functions. The reconstitution system of vesicle fusion with purified native vesicles, including LDCVs and synaptic vesicles, can be a good model for elucidating the molecular mechanisms by which cholesterol deficiency affects vesicle fusion^23^ (Fig. 1).

**Fig. 1:**
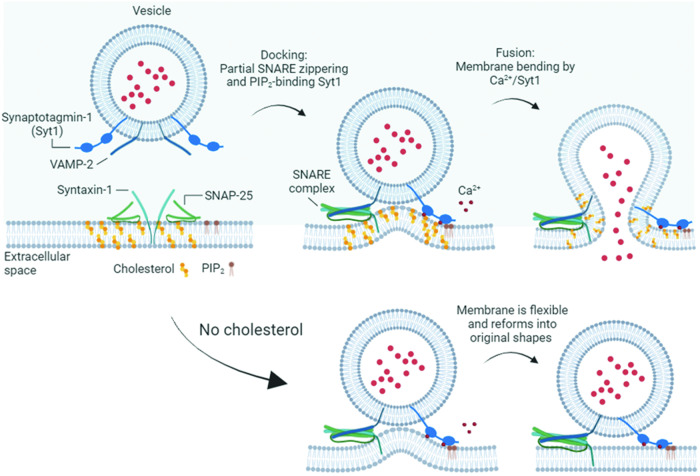
Schematic illustration of the roles of cholesterol in Ca^2+^-dependent vesicle fusion. Cholesterol is essential for Ca^2+^-dependent vesicle fusion. Synaptotagmin-1, a Ca^2+^ sensor that triggers fusion, induces local deformation of the plasma membrane. The plasma membrane is normally flexible and can return to its original shape due to membrane elasticity. However, cholesterol makes the membrane less fluid and more rigid, which helps to strengthen the membrane curvature and deformation, thus lowering the energy barrier for fusion. This image was created with BioRender.com.
  This reconstitution of vesicle fusion shows that cholesterol has little effect on Ca^2+^-independent basal fusion of synaptic vesicles but is required for Ca^2+^-dependent fusion of LDCVs and synaptic vesicles^23^. It is surprising that cholesterol reduction and imbalance specifically disrupt Ca^2+^-dependent vesicle fusion. Cholesterol has no effect on the membrane binding or insertion of synaptotagmin-1, a Ca^2+^ sensor for vesicle fusion^23^. Once synaptotagmin-1 is inserted into the membrane, cholesterol stabilizes and strengthens the local membrane bending and deformation induced by synaptotagmin-1^23^. The membrane is highly flexible, so it can reform into its original shape due to membrane elasticity^24^. Because cholesterol reduces membrane fluidity and increases membrane rigidity^25^, cholesterol can strengthen local membrane deformation and bending, thus lowering the energy barrier for Ca^2+^-dependent fusion^23^ (Fig. 1).
  Membrane bending and curvature play crucial roles in the process of vesicle fusion by lowering the energy barrier^26^. The energy stored in the curvature of the membrane can be released to facilitate the merging of two separate lipid bilayers^26–28^. For instance, smaller vesicles, which have a greater curvature, have greater bending energy per unit surface area, leading to a more efficient fusion process^26^. The insertion of proteins such as the C2AB domain of synaptotagmin-1 into the plasma membrane contributes to this curvature^29,30^, creating a high-energy state that can drive vesicle fusion.
  Cholesterol enhances membrane curvature and thus lowers the energy barrier for fusion^18^. It also strengthens local bending and deformation, particularly in the presence of Ca^2+^ and synaptotagmin-1, thereby driving Ca^2+^-dependent vesicle fusion^23^. Mechanical forces of membrane bending are critical for the dynamic process of vesicle fusion^31^, and cholesterol is an essential lipid for synaptic transmission because it strengthens membrane bending^23^.
  Synaptic transmission involves the release of neurotransmitters from synaptic vesicles into synapses, where neurotransmitters bind to receptors on postsynaptic neurons, thereby transmitting signals across the neural network^32^. When synaptic transmission is impaired due to disruptions in Ca^2+^-dependent vesicle fusion, neural network formation becomes impaired^33^. Disruption of Ca^2+^-dependent vesicle fusion and synaptic transmission via a cholesterol imbalance in the plasma membrane leads to reduced neural network activity and synaptic dysfunction and ultimately contributes to neurodegeneration. The loss of the neural network caused by the dysregulation of cholesterol homeostasis can result in declines in cognitive and motor functions associated with neurodegenerative diseases.
2. Membrane structure and protein clustering: cholesterol regulates membrane structure, fluidity, and curvature^25^. The plasma membrane is enriched in cholesterol^15^, which stabilizes membrane curvature and promotes vesicle fusion^17,25,34,35^. The membrane curvature and deformation stabilized by cholesterol bring the two membranes close together and enable fusion. Cholesterol also contributes to vesicle fusion by stabilizing fusion pores^25,36–38^. Therefore, cholesterol deficiency in neurons causes defects in membrane structure, resulting in neurodegeneration.
  Cholesterol also mediates the protein clustering involved in vesicle fusion. Exocytosis of neurotransmitter release is mediated by soluble *N*-ethylmaleimide-sensitive factor attachment protein receptor (SNARE) proteins^39,40^. Neuronal SNARE proteins consist of Q-SNARE in the plasma membrane (syntaxin-1 and SNAP-25) and R-SNARE in the vesicle membrane (synaptobrevin-2 or vesicle-associated membrane protein-2 (VAMP-2))^39^. Specialized microdomains within the plasma membrane, e.g., lipid rafts, detergent-resistant membranes, or liquid-ordered membrane microdomains, are enriched in cholesterol and concentrate signaling molecules^41–43^. Cholesterol plays an important role in the function and organization of SNARE proteins; syntaxin-1A, a neuronal SNARE protein, is concentrated in cholesterol-enriched domains in the plasma membrane^44,45^. The cholesterol-enriched membrane microdomains provide a specialized environment where syntaxin-1A interacts with its binding partners. In the context of synaptic transmission, the clustering of syntaxin-1A in cholesterol-enriched microdomains may enhance its interactions with other SNARE proteins, such as SNAP-25 and VAMP-2, to form the core SNARE complex necessary for vesicle fusion. This organization may influence the overall stability and efficiency of neurotransmitter release at the synapse, suggesting that cholesterol imbalance leads to defects in the clustering of the vesicle fusion machinery.
3. Oligomers of amyloid beta (Aβ) protein: cholesterol imbalance influences the aggregation and misfolding of proteins involved in neurodegenerative diseases, such as amyloid precursor protein (APP)^46^. The accumulation of Aβ plaques in the brain is a hallmark pathology of AD^47,48^. Aβ is derived from APP through enzymatic cleavage by the β-secretase Bace1^49^, and cholesterol modulates the processing of APP and the generation of Aβ^50,51^. High cholesterol levels can promote and accelerate the cleavage of APP by Bace1, resulting in increased Aβ production and aggregation, which contributes to the formation of toxic plaques^50–52^.
  Aβ40/Aβ42 peptides are the primary constituents of Aβ oligomers and plaques, which can be stabilized by biomolecules, including carbohydrates, nucleic acids, and lipids, e.g., cholesterol^53^. Extracellular cholesterol strengthens Aβ fibrils and oligomers against degradation by directly binding to Aβ^53,54^. Cholesterol has been implicated in the aggregation of Aβ peptides, particularly in the formation of Aβ oligomers^55–57^. Free cholesterol interacts with specific residues in Aβ peptides, particularly Phe19^55^. Cholesterol binds to the aromatic side chains of the Aβ peptide, thus increasing β-sheet formation in Aβ peptide oligomers^55^. A stable interaction between cholesterol and Phe19 leads to the formation of Aβ oligomers^55^, suggesting that the interaction of cholesterol with Aβ contributes to the formation of toxic Aβ oligomers, which play a critical role in AD pathology^57^.
  Cholesterol dramatically enhances and accelerates the onset of Aβ42 aggregation through a heterogeneous nucleation pathway^58^. Cholesterol imbalance and elevated extracellular levels of cholesterol can promote the production and accumulation of Aβ peptides, which induce the formation of Aβ oligomers in the brain, thus contributing to neuronal damage and cognitive decline^57^ (Fig. 2). Aβ monomers misfold and form β-sheet-rich oligomers that eventually impair synaptic plasticity and neuronal survival^59^. The direct interaction of cholesterol with Aβ can stimulate and activate toxic Aβ oligomerization, which is a critical factor in AD pathogenesis.

**Fig. 2:**
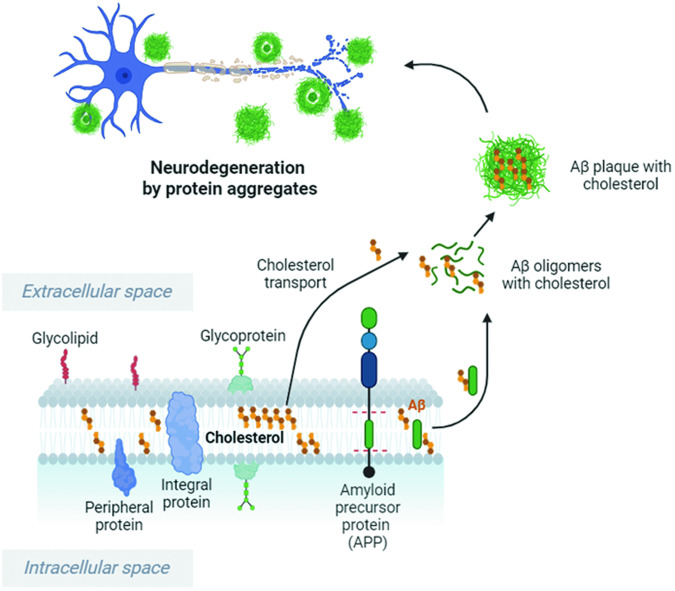
Schematic overview of cholesterol transport to promote Aβ aggregation for neurodegeneration. Cholesterol enhances and accelerates APP cleavage by Bace1, leading to increased Aβ oligomer and plaque formation. Cholesterol binds to Aβ and increases the resistance of Aβ fibrils and oligomers to degradation. Cholesterol imbalance and high extracellular cholesterol levels can stimulate the production and accumulation of Aβ peptides, which cause Aβ oligomer formation and aggregation in the brain, resulting in neuronal damage. This image was created with BioRender.com.
  The membrane curvature induced by cholesterol might contribute to Aβ aggregation^60^. High membrane curvature promotes Aβ nucleation, accelerating amyloid fibril formation^61^. Aβ aggregates can readily form on membranes with high curvature^62^. High membrane curvature, such as that associated with lipid rafts and membrane budding processes, might provide favorable conditions for the nucleation of Aβ peptides^61–63^. Given that cholesterol stabilizes high membrane curvature, cholesterol-mediated curvature of membranes may lead to altered lipid packing that engages Aβ hydrophobic groups and promotes Aβ fibrillar structures^64^. Lipid packing defects that occur in curved membranes may induce conformational changes in Aβ peptides, thus promoting Aβ fibrils and aggregation^64^. This aggregation is a hallmark of AD, and understanding the underlying mechanisms of membrane curvature is crucial for developing potential therapeutic strategies. The interplay between membrane curvature and Aβ aggregation is complex, and ongoing research continues to unveil the molecular details involved.
4. Tau aggregation: while Aβ oligomers and aggregation are primarily associated with AD, another hallmark of AD is the aggregation of hyperphosphorylated tau proteins into neurofibrillary tangles (NFTs)^65^. Tau is a cytoskeletal protein that stabilizes microtubules in neurons, but it is hyperphosphorylated in AD^66^. The interaction between tau proteins and cell membranes, particularly in cholesterol-rich regions, is a critical factor in the tau aggregation process^67,68^. The membrane binding of tau can induce conformational changes and aggregation with β-sheet-rich structures^69^. NFTs in AD brains contain cholesterol^54,70^, which could modulate tau-membrane interactions and affect tau aggregation^68,71^.
  As cholesterol can influence membrane curvature, high membrane curvature caused by cholesterol can induce changes in the conformation of tau proteins and promote tau aggregation^72^. Membranes with high curvature that contain cholesterol induce tau fibril formation, whereas cholesterol depletion abolishes tau fibril formation^72^. Cholesterol-free membranes fail to induce the formation of tau fibrils, suggesting that cholesterol-mediated tau aggregation is essential for the pathology of tauopathies^72^. Membrane morphologies result in different hydrophobic interactions that lead to the β-sheet structures of tau proteins^72^. The association of tau with highly curved membranes is initiated by electrostatic attraction between the Lys sidechains of tau and the lipid headgroups; cholesterol might further strengthen this electrostatic attraction for tau fibril formation^72^. However, how cholesterol facilitates tau nucleation remains a topic of further study, and understanding the underlying molecular mechanisms is crucial for developing therapeutic strategies involving the disruption of tau aggregation.
5. α-Synuclein (α-syn) aggregation: a characteristic feature of PD is the accumulation of misfolded α-syn proteins in Lewy bodies (LBs)^73^. The interaction between α-syn and lipids is important for fibril formation, and the aggregation of α-syn is induced by binding to membrane lipids^74^. Lipids dramatically enhance the primary nucleation of α-syn to form aggregates associated with neurodegeneration^74^.

**Table 1:** Cholesterol and neurodegenerative diseases.

Neurodegenerative disease	Relation to cholesterol	Refs.
Alzheimer’s Disease	High cholesterol promotes the cleavage of APP by Bace1, resulting in Aβ production and aggregation.	^50–52^
	Cholesterol is the primary constituent of Aβ oligomers and plaques.	^53^
	Cholesterol strengthens Aβ fibrils and oligomers against degradation by directly binding to Aβ.	^53,54^
	Cholesterol leads to the formation of Aβ oligomers through interaction with Phe19 of Aβ, thus stabilizing Aβ oligomers.	^55–57^
	Membrane curvature induced by cholesterol accelerates Aβ aggregation.	^60–64^
	ApoE4, a major risk factor for AD, is associated with dysregulation of cholesterol transport.	^46^
	Cholesterol stimulates tau aggregation process through tau conformational changes into β-sheet-rich structures.	^54,67–71^
	Cholesterol-containing membranes with high curvature induce tau fibril formation.	^72^
Parkinson’s Disease	Cholesterol is a component of LBs together with α-syn.	^75^
	Cholesterol accelerates α-syn aggregation and LB formation.	^76^
	Cholesterol induces toxic α-syn oligomers and fibrils by forming β-sheet structures.	^78^
Huntington’s Disease	Mutant huntingtin (mHTT) aggregates in HD and reduces nuclear translocation of the sterol regulatory element-binding protein 2 (SREBP2).	^85^
	Cholesterol modulates mHTT aggregation by regulating membrane interactions.	^86,87^
Multiple Sclerosis (MS)	Disrupted cholesterol metabolism is associated with MS, and cholesterol induces misfolded protein aggregation.	^88^

Together with α-syn, cholesterol, which is a component of LBs^75^, accelerates α-syn aggregation and LB formation^76^. Cholesterol interacts with α-syn, and high cholesterol levels can promote α-syn aggregation^76,77^, leading to the formation of toxic LBs, which contributes to neurodegeneration in PD.

α-Syn binds to membranes through electrostatic interactions and hydrogen bonding, but cholesterol reduces the coulomb interactions and hydrophobic interactions between α-syn and membranes^78^. Cholesterol decreases lipid packing defects and lipid fluidity, thereby dysregulating the membrane binding of α-syn^78^; membrane-bound α-syn can have a β-sheet structure that induces the formation of toxic α-syn oligomers and fibrils. Together, the imbalance and dysregulated distribution of cholesterol in neurons cause neurodegeneration by accelerating α-syn aggregation and LB formation.

### Possible therapeutic approaches for cholesterol imbalance

The apolipoprotein E (ApoE) gene is involved in the metabolism and transport of cholesterol^79,80^. There are three main variants or alleles of the APOE gene, namely, ApoE2, ApoE3, and ApoE4^81^. The ApoE4 protein is the most important risk factor for late-onset AD^82^, i.e., the most common form of the disease that occurs after age 65. The molecular mechanisms by which ApoE4 contributes to AD are complex and not fully understood, but ApoE4 likely promotes Aβ aggregation by transporting cholesterol^79,83^. ApoE4 can induce cholesterol imbalance by transporting cholesterol from the plasma membrane in neurons to protein aggregates (Fig. 2). Given that cholesterol imbalance causes neurodegeneration, ApoE4 may be a possible target for mitigating Aβ aggregation and treating cholesterol imbalance.

Overall, the maintenance of cholesterol homeostasis is important for preventing or slowing the pathogenesis of neurodegenerative diseases. Strategies aimed at regulating cholesterol levels or targeting cholesterol-mediated pathways involved in protein aggregation might be potential therapeutic approaches to treating neurodegenerative diseases, although neurodegenerative diseases are complex and cholesterol imbalance is one of many contributing factors^11,84^.

## Conclusion

Cholesterol is an essential component of the body that helps maintain the integrity of cell membranes. The role of cholesterol dysregulation in neurodegeneration is an active area of research, and the precise mechanisms involved may vary depending on the specific condition. The balance between the beneficial and detrimental effects of cholesterol remains complex. An imbalance in cholesterol regulation is a common feature of neurodegenerative conditions such as AD and PD. Therapeutic approaches for modulating cholesterol metabolism or targeting specific cholesterol-related pathways could be potential strategies for mitigating neurodegeneration. While cholesterol-lowering drugs, e.g., statins, have shown some potential in reducing the risk of certain neurodegenerative diseases, further research is required to fully understand the role of cholesterol and develop targeted therapeutic interventions.
